# Supplementary material for: Interrater reliability of novice examiners using A-mode ultrasound and skinfolds to measure subcutaneous body fat
Source: PLoS One. 2020 Dec 14;15(12):e0244019. doi: 10.1371/journal.pone.0244019 (PMC7735566; doi:10.1371/journal.pone.0244019)
Supplement: Attachment — Submitted filename: Plos One_peer review_10.27.2020.docx [file pone.0244019.s001.docx]

Overall

This is interesting work that has great practical implications for fitness professionals or clinicians. The interrater reliability for two body composition techniques, one being widely used, that are easily accessible for assessing body composition were compared. The manuscript is written well and has great potential for aiding practitioners, but a couple of additional analyses and methodological details would improve the utility of this information.

Abstract

N/A

Introduction

N/A

Methods

Line 81: Considering the nature of this work and importance of familiarity, it would be helpful if there was at least a rough approximation of the average experience (e.g., < 1 hr?) for these students with the SKF technique

What was done to limit compression via the US probe and keep it consistent? It is well known that this influences the morphology of the tissue deep to the probe, so this is at least worth mentioning. Differences in compression could arguably be one of the larges influences on interindividual variability.

A limitation the unknown equation that is used for the US, especially since this means the two techniques are likely using different equations to calculate BF%

For a more comprehensive examination of reliability, the standard error of measurement (SEM) should be provided for both modalities. The SEM provides a different analysis of reliability and is more useful for practitioners. See Weir, 2005.

Given the practical utility of this study and its findings, it would be much improved if the minimal difference to be considered real was calculated (aka MD). This could be very useful for practitioners to know, depending on the testing modality, what a meaningful change would be.

A figure indicating a typical US scan and the selected subcutaneous fat would helpful, especially considered the feasibility for novices to use this technique.

Results

Line 13: “accumulated” indicates there was an intervention or that this was a longitudinal study.

Discussion

Based on a qualitative inspection of the scatterplots, it looks the greatest interindividual differences were at relatively higher fat levels. Was this a common observation? While this is to be expected, it is worth mentioning as it could be useful for practitioners to be aware of.
