# Supplementary material for: Interrater reliability of novice examiners using A-mode ultrasound and skinfolds to measure subcutaneous body fat
Source: PLoS One. 2020 Dec 14;15(12):e0244019. doi: 10.1371/journal.pone.0244019 (PMC7735566; doi:10.1371/journal.pone.0244019)
Supplement: Attachment — Submitted filename: Response to Reviewers.docx [file pone.0244019.s002.docx]

**We thank the reviewers for their time and careful reading of our manuscript. Our responses are listed in bold below each of the comments.**

Reviewer #1

Overall

This is interesting work that has great practical implications for fitness professionals or clinicians. The interrater reliability for two body composition techniques, one being widely used, that are easily accessible for assessing body composition were compared. The manuscript is written well and has great potential for aiding practitioners, but a couple of additional analyses and methodological details would improve the utility of this information.

**Thank you.**

Abstract

N/A

Introduction

N/A

Methods

Line 81: Considering the nature of this work and importance of familiarity, it would be helpful if there was at least a rough approximation of the average experience (e.g., < 1 hr?) for these students with the SKF technique

**Added (line 83)**

What was done to limit compression via the US probe and keep it consistent? It is well known that this influences the morphology of the tissue deep to the probe, so this is at least worth mentioning. Differences in compression could arguably be one of the larges influences on interindividual variability.

**Students were instructed to not apply pressure as part of the classroom instruction; however, as mentioned in the manuscript, students took both US and SKF measurements without any feedback (line 112-113). We added a sentence to the discussion to highlight your concern (line 250-252).**

A limitation the unknown equation that is used for the US, especially since this means the two techniques are likely using different equations to calculate BF%

**Correct, the measurement sites were the same for the US and SKF measurements, but the manufacturer’s conversion to %BF for the US measurement is proprietary (line 131-134). This might be more of a concern (limitation) if the intent was to compare the %BF estimations between SKF and US. However, it doesn’t really matter if the SKF and US formulas do not produce the same %BF estimation in this study, as we were looking at interrater reliability (or the consistency of measures between two examiners).**

For a more comprehensive examination of reliability, the standard error of measurement (SEM) should be provided for both modalities. The SEM provides a different analysis of reliability and is more useful for practitioners. See Weir, 2005.

**SEM data were added (line 143 and 177-178). RMSE was deleted, as this is somewhat redundant with SEM.**

Given the practical utility of this study and its findings, it would be much improved if the minimal difference to be considered real was calculated (aka MD). This could be very useful for practitioners to know, depending on the testing modality, what a meaningful change would be.

**Added (line 144 and 179-184)**

A figure indicating a typical US scan and the selected subcutaneous fat would helpful, especially considered the feasibility for novices to use this technique.

**Added; cited (line 118); added (line 123-124)**

Results

Line 13: “accumulated” indicates there was an intervention or that this was a longitudinal study.

**Changed (line 151)**

Discussion

Based on a qualitative inspection of the scatterplots, it looks the greatest interindividual differences were at relatively higher fat levels. Was this a common observation? While this is to be expected, it is worth mentioning as it could be useful for practitioners to be aware of.

**No. As you stated, heteroscedasticity is somewhat expected with the SKF method (and possibly exists with the US method as well, but there are too few studies with obese participants to say for certain). However, that was not observed here. The greater interindividual difference with the SKF method compared to the US method is clear, but there does not appear to be increased variability with increased fatness for either method in this sample.**

Reviewer #2

This topic is of important to athletic trainers, personal trainers, coaches etc, as field methods are generally used to measure body composition. Overall, it is a very well designed study and well written.

**Thank you.**

Does the study have a hypothesis?
**Hypothesis added (line 76-77).**

Line 45: Need more than one reference to support this statement.

**An additional sentence (and reference) was added to support this statement (line 46-47).**

Methods:
Please provide a power analysis. What was the p value set to for significance?
**A formal a priori power analysis was not done because our sample size was predetermined (limited to students enrolled in the course). Further, it is inappropriate to report post-hoc observed power (see O’Keefe. Communication Methods and Measures. 2007;1:291-299. and Hoenig & Heisey. The American Statistician. 2001;55:19-24.). However, our sample size (N = 80) is substantially larger than previously published A-mode ultrasound body composition research that reported interrater reliability (N = 31 for Hendrickson et al. 2019; N = 45 for Wagner et al. 2016), and it was also larger than studies that included test-retest reliability of this method (N = 11 for Loenneke et al. Clinical Physiology and Functional Imaging. 2014;34:159-162. and N = 47 for Smith-Ryan et al. Plos One. 2014;e91750.).**

**Intraclass correlation coefficients (ICC) are evaluated with 95% confidence intervals rather than p-values.**

Discussion:
Future research? What are the limitations and strengths of this study?

**A paragraph was added (line 264-270) before the conclusion to address these questions.**
